# PDZ domain-binding motif of human T-cell leukemia virus type 1 Tax oncoprotein is essential for the interleukin 2 independent growth induction of a T-cell line

**DOI:** 10.1186/1742-4690-2-46

**Published:** 2005-07-23

**Authors:** Chikako Tsubata, Masaya Higuchi, Masahiko Takahashi, Masayasu Oie, Yuetsu Tanaka, Fumitake Gejyo, Masahiro Fujii

**Affiliations:** 1 Division of Virology, Niigata University Graduate School of Medical and Dental Sciences, 1-757 Asahimachi-Dori, Niigata 951-8510, Japan; 2 Division of Clinical Nephrology and Rheumatology, Niigata University Graduate School of Medical and Dental Sciences, 1-757 Asahimachi-Dori, Niigata 951-8510, Japan; 3 Department of Infectious Disease and Immunology, Okinawa-Asia Research Center of Medical Science, Faculty of Medicine, University of the Ryukyus, Okinawa, Japan

## Abstract

**Background:**

Human T-cell leukemia virus type 1 (HTLV-1) is the etiologic agent of adult T-cell leukemia (ATL), whereas HTLV type 2 (HTLV-2), is not associated with ATL or any other leukemia. HTLV-1 encodes the transforming gene *tax1*, whose expression in an interleukin (IL)-2-dependent T-cell line (CTLL-2) induces IL-2-independent growth.

**Results:**

In this study, we demonstrated that IL-2-independent growth induction by Tax1 was abrogated by mutations of the PDZ domain-binding motif (PBM) at the Tax1 C-terminus. HTLV-2 Tax2, which shares 75% amino acid identity with Tax1 but does not have a PBM, was not able to induce IL-2-independent growth of CTLL-2.

**Conclusion:**

Our results suggest that Tax1, through interaction with PDZ domain protein(s) induces IL-2-independent growth, which may be a factor in multi-step leukemogenesis caused by HTLV-1.

## Findings

Adult T-cell leukemia (ATL) is an extremely aggressive T-cell leukemia [1,2], and it is characterized by malignant expansion of CD4 positive T-cells infected with human T-cell leukemia virus type 1 (HTLV-1). HTLV-1 is an onco-retrovirus, which immortalizes human CD4 T-cells *in vitro *[3,4]. Such an immortalization event is, however, not sufficient for ATL development, since a minority of HTLV-1-infected individuals (~5%) suffer ATL 60 years on average after the infection [2,5,6]. Accumulating evidence suggests that genetic and epigenetic changes in HTLV-1-infected T-cells and deterioration of host immune activities are prerequisites for ATL development [2]. HTLV type 2 (HTLV-2) is molecularly and biologically similar to HTLV-1 [7,8]. HTLV-2 also immortalizes primary human T-cells with equivalent efficiency to HTLV-1, although HTLV-2 preferentially immortalizes CD8 T-cells [9]. Regardless of such similarities, HTLV-2 is not associated with ATL or any other leukemia [10]. Thus, HTLV-2 can not promote multi-step leukemogenesis. However, the underlying mechanism by which HTLV-1 promotes multi-step leukemogenesis has not yet been elucidated.

HTLV-1 and HTLV-2 encode functionally and structurally similar proteins, Tax1 and Tax2, respectively [7,11,12], and they are candidate factors responsible for distinct pathogenic activities of the two viruses. Tax1 and Tax2 were originally identified as transcriptional activators of their own gene expression [11,12]. Later they were shown to play crucial roles in the immortalization of T-cells [13,14]. Tax1 by itself immortalizes primary human T-cells in an interleukin (IL)-2-dependent manner [15,16]. Tax1 inhibits several modes of apoptosis [17], and stimulates the cell cycle progression in primary T-cells as well as in T-cell lines [18,19]. In addition, in transgenic animals Tax1 induces various malignancies such as fibrosarcoma and natural killer cell leukemia [20,21]. Consistent with the above activities, recombinant HTLV-1 and HTLV-2 carrying inactive *tax1 *and *tax2 *genes, respectively, cannot transform primary human T-cells [13,14]. Evidence suggests that the activation of cellular genes by Tax1 is essential for T-cell immortalization [22]. For instance, Tax1 activates the expression of genes encoding cytokines, cytokine receptors, chemokines, cell cycle regulators and anti-apoptotic factors [22-31]. Tax1 and Tax2 generally activate the same sets of cellular genes with equivalent efficiency, although some differences have been reported.

**Figure 1:**
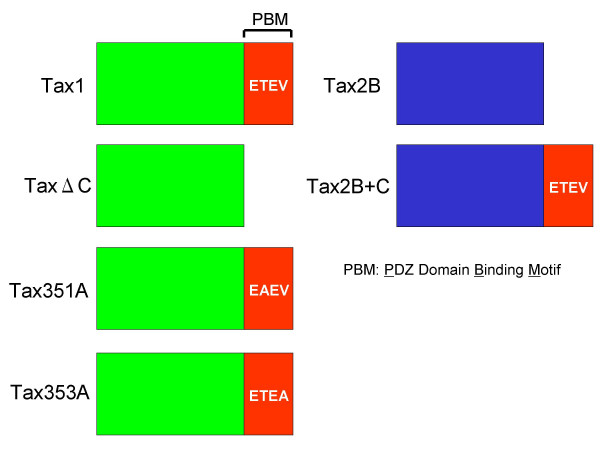
**Structure of Tax1, Tax2B and their mutant proteins**. The amino acid sequence of PBM and its mutants are indicated. The *tax1*, *tax2B *genes and their mutant genes inserted in the pHβ Pr-1-neo expression plasmid have been described previously [33]. To convert the expression plasmid from pHβPr-1-neo to pβA-IRESpuro plasmid (pβAIP), an *Eco*RI-*Bam*HI fragment containing the β-actin promoter of pHβPr-1-neo was inserted into the *Nru*I-*Bam*HI site of pIRESpuro3 (BD Biosciences) by a blunt-end ligation. Then, wild type *tax *or *tax *mutant cDNAs were inserted into the *Bam*HI site of pβAIP.

**Figure 2:**
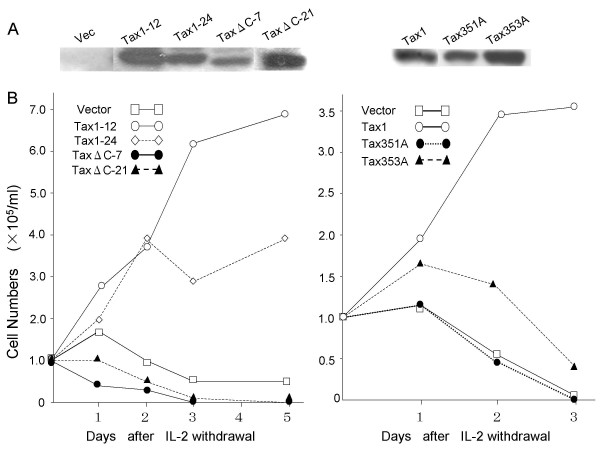
**PBM is essential for IL-2-independent growth of CTLL-2 cells induced by Tax1**. (A) CTLL-2 is a mouse T-cell line. This cell line was cultured in RPMI1640 medium supplemented with 10% heat-inactivated fetal bovine serum (RPMI-FBS), antibiotics and 0.5 nM recombinant human IL-2. To establish CTLL-2 cell lines expressing Tax or Tax mutant proteins, CTLL-2 cells (1 × 10^7^) were suspended in 400 μl Opti MEM1 (Gibco BRL, Gaithersburg, MD), mixed with 20 μg of the vector plasmid (pβAIP) or with expression plasmids encoding Tax1 or Tax mutants, and then pulsed at 200 V and 975 F. The cells were seeded in 96 well plates 24 h after electroporation and cultured in RPMI-FBS containing 0.5 nM IL-2 and 2 μg/ml puromycin for 4 to 6 weeks. Puromycin-resistant cells were screened for the expression of Tax protein by Western blot analysis using mouse anti-Tax1 monoclonal antibody (TAXY-7) [42] as described previously [33]. (B) CTLL-2/Vector, and two of each CTLL-2 clone expressing Tax1 or Tax mutant proteins were washed twice with phosphate-buffered saline (PBS) and cultured in IL-2-free medium for 3–5 days. Cell growth was measured by the trypan blue staining method using light microscopy.

We previously found that Tax1 and Tax2 transform a rat fibroblast cell line (Rat-1) to induce colonies in soft agar (CFSA, colony formation in soft agar), and the activity of Tax1 is greater than that of Tax2 [32]. The experiments using their chimeric proteins indicated that the PDZ domain-binding motif (PBM) located at the Tax1 C-terminus, S/TXV (S/T, serine or threonine; X, any amino acid; V, valine), is responsible for the high CFSA activity relative to Tax2 [33]. Through this motif, Tax1 but not Tax2 was found to bind to PDZ domain-containing proteins, including Dlg, a mammalian homologue of Drosophila discs large tumor suppressor [33-36]. These results present an attractive hypothesis that PBM is a factor responsible for the distinct pathogenic activities of HTLV-1 and HTLV-2. Since HTLV-1 is a T-cell-tropic virus, in this study, we examined the activity of Tax1 PBM in T-cells. To do this, we used several mutant genes that had been previously characterized (Figure 1 and Table 1) [33]. The TaxΔC gene contains a C-terminal four-amino acid deletion abrogating PBM in Tax1. Tax351A and Tax353A are substitution mutants of Tax1 PBM, at amino acids 351 and 353 in Tax1, respectively. These three PBM mutants did not interact with PDZ domain-containing proteins such as Dlg and MAGI-3 [33,36]. Tax2B+C is a chimeric Tax2B gene with a wild type Tax1 PBM peptide, and Tax2B+C but not Tax2B interacts with Dlg. These genes in pβA-IRES-puro plasmid (pβAIP) were transfected into an IL-2 dependent mouse T-cell line (CTLL-2), and the cells were then selected by puromycin in the presence of IL-2. Western blotting analysis using anti-Tax1 antibody showed that two independent TaxΔC clones (TaxΔC-7, TaxΔC-21) and two independent Tax1 clones (Tax1-12, Tax1-24) expressed TaxΔC and Tax1 protein, respectively. The amounts of mutant Tax1 protein relative to wild type protein were generally equivalent, and TaxΔC-21 cells expressed the Tax protein higher than Tax1-24 cells (Figure 2). These characterized cells were then cultured in the absence of IL-2 for 3–5 days. CTLL-2 cells transfected with a control plasmid (pβAIP) did not grow in the absence of IL-2, and most of the cells died approximately 3 days after IL-2 withdrawal, whereas two CTLL-2 clones transfected with wild-type *tax1 *plasmid continued to grow for 5 days. This was consistent with previous findings that stable Tax1 expression in CTLL-2 conferred a permanent IL-2-independent growth phenotype [37]. On the other hand, two CTLL-2 clones transfected with *taxΔC *did not grow in the absence of IL-2, and were close to cell death approximately 2 days after IL-2 withdrawal. In addition, two PBM mutant clones, CTLL-2/Tax351A and CTLL-2/Tax353A, did not grow in the absence of IL-2. These results show that Tax1 PBM is essential for IL-2-independent growth induction of CTLL-2 cells. Unexpectedly, in spite of three independent trials, we could not establish CTLL-2 cells expressing Tax2B or Tax2B+C even in the presence of IL-2. Although anti-Tax1 and anti-Tax2 antibodies did not detect Tax2B and Tax1, respectively, previous study using a chimeric Tax1 with Tax2B showed that the amounts of Tax1 and Tax2B proteins were expressed equivalently in the cells [32]. Thus, these results suggested that Tax2B has a toxic effect in CTLL-2 cells.

**Table 1:** Characterization of Tax1, Tax2B, and their mutants

	Tax	Tax△C	Tax351A	Tax353A	Tax2B	Tax2B+C
IL-2 independent proliferation of CTLL-2	+	-	-	-	-	-
% Outgrowth	30–40	3–5	2–5	1–4	0	0
Dlg binding^#^	++	-	-	-	-	++
CFSA of Rat-1^#^	+++	+	+	+	+	+++

^#^The results from reference 33.

**Figure 3:**
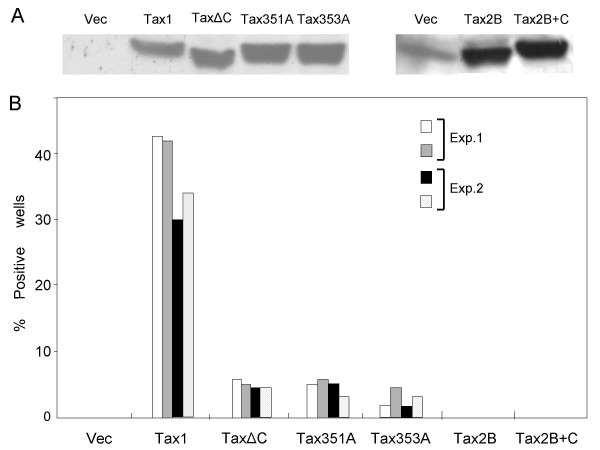
**PBM is essential for outgrowth of CTLL-2/Tax cells in the absence of IL-2**. (A, B) CTLL-2 cells (10^7^) were transfected either with the vector plasmid (pHβPr-1-neo) or with expression plasmids encoding Tax1, Tax2B or their mutants by electroporation. The cells were divided into two groups 24 h after transfection. From the first group, living cells were collected using Ficoll-Paque Plus (Amersham Biosciences) and used for Western blot analysis (A) using anti-Tax1 antibody (TAXY7) or anti-Tax2B polyclonal antibody [43]. The second group (B) was seeded into 96 well plates and cultured in RPMI-FBS without IL-2 for 3 weeks, and the number of IL-2-independent colonies was counted under light microscopy. The percentage of positive wells indicates the proportion of the wells containing outgrowth of CTLL-2 cells. The data relates to two independent experiments with each duplicated transfection.

To confirm the role of PBM in IL-2 independent growth of CTLL-2, we transfected *tax1*, *tax2B *or their mutant plasmids into CTLL-2 cells, and the cells were seeded onto 96-well plates at a density of 1 × 10^4 ^cells/well, and cultured without IL-2 for three weeks. CTLL-2 cells transfected with the *tax1 *plasmid induced visible cell colonies in about 40% of the wells. This was due to the expression of Tax1 protein, since such cell growth was not observed in any wells containing CTLL-2 transfected with the empty vector plasmid. CTLL-2 cells transfected with three Tax1 PBM mutants also induced IL-2-independent cell growth, but the number of positive wells and the number of cells in each well were much lower than those of CTLL-2/Tax1 (Figure 3B, data not shown). Moreover, while CTLL-2/Tax1 cells continued to grow in the absence of IL-2 for at least two months, none of CTLL-2/TaxΔC cells grew any further (data not shown). This weakened activity of Tax1 PBM mutants was not due to their expression level in CTLL-2 cells, since Western blot analysis using anti-Tax1 antibodies detected equivalent expression relative to Tax1 after transfection (Figure 3A). These results indicate that TaxΔC still has IL-2-independent growth inducing activity in CTLL-2 cells, but the activity is much less than that of Tax1. Both Tax2B and Tax2B+C are completely devoid of such activity, further indicating that Tax2B does not have IL-2-independent growth inducing activity in CTLL-2 cells.

HTLV-1 Tax1 oncoprotein changes the cell growth of CTLL-2 from being IL-2-dependent to being IL-2-independent [37]. In this study, we showed that the PBM of Tax1 is essential for this activity in CTLL-2. Unlike Tax1, HTLV-2 Tax2 did not induce IL-2-independent growth, consistent with the absence of PBM in Tax2. Taken together with the strict conservation of PBM only in HTLV-1 Tax1 [33], these results suggest that HTLV-1 and HTLV-2 infection have distinct activity to growth of infected T-cells, and such a difference may be a factor responsible for ATL development.

Tax1, but not Tax2, interacts with the PDZ domain containing proteins Dlg and MAGI-3 [33,36]. Cotransfection and immunoprecipitation experiments showed that the three Tax1 mutants used here are severely defective in interaction with both Dlg and MAGI-3 proteins (Table 1). Dlg is highly expressed in T-cells including HTLV-1-infected T-cell lines [33,35], whereas MAGI-3 was detected only by reverse-transcription polymerase chain reaction analysis [36]. Since Dlg is a tumor suppressor gene product in Drosophila, it is an attractive candidate to play a role in IL-2-independent growth induction in CTLL-2 cells. It should be noted that there are many PDZ domain-containing genes in human. Thus, it is important to consider such proteins as candidates to mediate Tax1 activity in HTLV-1-infected T-cells.

We recently showed that Tax2, through the activation of transcription factor NFAT, constitutively induces the expression of IL-2, and the induced IL-2 promotes the cell growth of HTLV-2-infected T-cell lines, whereas such autocrine growth stimulation was not detected in HTLV-1-infected T-cell lines [38]. Tax2, however, did not induce IL-2-independent growth of CTLL-2 cells. In addition to Tax2B, Tax2B+C also failed to induce IL-2-independent growth of CTLL-2 cells. Tax2B+C, but not Tax2, transforms Rat-1 cells (CFSA) to the same extent as Tax1, and interacts with Dlg and MAGI-3 (Table 1) [33,36]. Thus, the binding of Tax2B+C to PDZ domain-containing proteins is not sufficient to induce IL-2-independent growth. Although it is unclear why we could not detect the activity of Tax2 to induce IL-2-independent growth, one possibility is that NFAT activation by Tax2 may induce the expression of pro-apoptotic genes such as Fas ligand, which may induce apoptosis of CTLL-2 cells, thereby masking the growth-promoting effect of IL-2.

Tax1 PBM plays crucial roles in the growth promoting activities in two different cell backgrounds; IL-2-independent growth induction of a T-cell line and transformation (CFSA) of a Rat-1 fibroblast cell line (Table 1), but it is unclear whether these two activities utilize the same mechanism. Both the number and size of the transformed colonies of Rat-1/Tax1 cells were greater than those of Rat-1/Tax2B cells, but the presence of Tax1 PBM was only correlated with the number but not the colony size [33]. These results suggest that the Tax1 PBM may have a selective role in the initiation of anchorage-independent growth of Rat-1 cells in soft agar but not the subsequent growth speed. Similarly, Tax1 PBM might be required for the initial cell growth of CTLL-2 deprived from IL-2, but not the subsequent rate of growth. Further analysis is required to solve this interesting question.

Several tumor viruses have both high-risk and low-risk subtypes. High-risk viruses induce malignancies such as cancers or leukemia in the host, whereas low-risk viruses induce benign tumors or lymphoproliferative diseases. Human papilloma virus (HPV) is such a virus, and only high-risk subtypes are associated with cervical cancers. Interestingly, an E6 oncoprotein of high-risk HPVs also contains PBM, and the motif is associated with high level of transforming activities measured by CFSA or focus formation of fibroblast cell lines *in vitro *[39]. Moreover, while E6 induces tumors in transgenic mice, deletion of the E6 PBM abrogates such activity [40]. Thus, PBM is a common determinant for high-risk oncoviruses, thereby being a useful tool for elucidating the molecular mechanism of malignant conversion of virus-infected cells.

Several inhibitors of transcription factor NF-κB induced apoptosis in HTLV-1-infected T-cell lines [41]. In addition, activation of NF-κB by Tax1 was well correlated with the induction of IL-2-independent growth of CTLL-2 [37]. However, NF-κB does not account for cell death of CTLL-2/TaxΔC cells in the absence of IL-2, since TaxΔC has equivalent NF-κB activity to Tax1 [36]. Taken together, the present results suggest that Tax1 PBM cooperates with NF-κB to induce IL-2-independent growth of HTLV-1-infected cells.

## Competing interests

The author(s) declare that they have no competing interests.

## Authors' contributions

CT, MH and MT carried out the establishing the cell lines and the functional analysis of the cell lines. MO, YT, FG and MF participated in the experimental design, data interpretation, and writing of the manuscript.
